# Multidisciplinary Management of a Giant Plexiform Neurofibroma by Double Sequential Preoperative Embolization and Surgical Resection

**DOI:** 10.1155/2013/987623

**Published:** 2013-03-28

**Authors:** Roberto Vélez, Sergi Barrera-Ochoa, David Barastegui, Mercedes Pérez-Lafuente, Cleofe Romagosa, Manuel Pérez

**Affiliations:** ^1^Orthopaedic Oncology Unit, Orthopaedic Surgery and Traumatology Department, Hospital Universitari Vall d'Hebron, Pg Vall d'Hebron 129-139, 08035 Barcelona, Spain; ^2^Orthopaedic Surgery and Traumatology Department, Hospital Universitari Vall d'Hebron, Pg Vall d'Hebron 129-139, 08035 Barcelona, Spain; ^3^Interventional Radiology Service, Radiology Department, Hospital Universitari Vall d'Hebron, Pg Vall d'Hebron 129-139, 08035 Barcelona, Spain; ^4^Pathology Department, Hospital Universitari Vall d'Hebron, Pg Vall d'Hebron 129-139, 08035 Barcelona, Spain

## Abstract

Plexiform neurofibromas are benign tumors originating from subcutaneous or visceral peripheral nerves, which are usually associated with neurofibromatosis type 1. Giant neurofibromas are very difficult to manage surgically as they are extensively infiltrative and highly vascularized. These types of lesions require complex preoperative and postoperative management strategies. This case report describes a 22-year-old female with a giant plexiform neurofibroma of the lower back and buttock who underwent pre-operative embolization and intraoperative use of a linear cutting stapler system to assist with haemostasis during the surgical resection. Minimal blood transfusion was required and the patient made a good recovery. This case describes how a multidisciplinary management of these large and challenging lesions is technically feasible and appears to be beneficial in reducing perioperative blood loss and morbidity. Giant neurofibroma is a poorly defined term used to describe a neurofibroma that has grown to a significant but undefined size. Through a literature review, we propose that the term “giant neurofibroma” be used for referring to those neurofibromas weighing 20% or more of the patient's total corporal weight.

## 1. Introduction

Neurofibromatosis type 1 (NF1) is an autosomal dominant genetic syndrome caused by mutations in genes coding for neurofibromin. NF1 is one of the most common human genetic diseases. The incidence of NF1 has been estimated to be between 1 and 3000–4000 [1, 2] and affects male and female subjects equally in all races. This mutation predisposes patients to the development of multiple neurofibromas [3]. Neurofibromas are common, representing approximately 5% of all benign soft-tissue tumors in large surgical series. Three types of neurofibromas are classically described: localized, diffuse, and plexiform [4–6]. 

Plexiform neurofibromas (PNFs) rarely grow to be larger than 5 cm; however, neurofibromas can undergo continuous enlargement and eventually become giant lesions. Early childhood, puberty, and childbearing age are considered to be the periods of greatest risk for disease progression. Furthermore, PNFs have a potential for transformation into highly malignant peripheral nerve sheath tumors, which occur in approximately 5% of patients [7].

Giant neurofibroma is a poorly defined term used to describe a neurofibroma that has grown to a significant but undefined size. There are a number of case reports and series found in the literature discussing giant neurofibromas [7–16].

PNFs are difficult to manage surgically as they are extensively infiltrative, highly vascularized and tend to recur. Surgical treatment must be decided judiciously and individualized for each patient [5]. Major complications of neurofibromas include malignant differentiation [4, 17] and potentially life-threatening hemorrhage [18] fortunately, these are quite rare. On the other hand, minor complications such as local infections or wound problems are very common. 

We report a case of a giant PNF of the lower back and buttock. It was managed with double sequential intravascular embolization followed by surgical resection, demonstrating that this technique is technically feasible and should be considered for these lesions. Additionally, through a literature review, we propose a new definition of giant neurofibroma.

## 2. Case Report

An otherwise healthy 22-year-old caucasian woman consulted for an initial orthopedic examination due to an accelerated growth of a large mass over the posterior aspect of her right buttock and lower lumbar region. The mass had been present for over 5 years and had gradually enlarged during this time. Recently, the lesion had increased in size rather rapidly.

**Figure 1:**
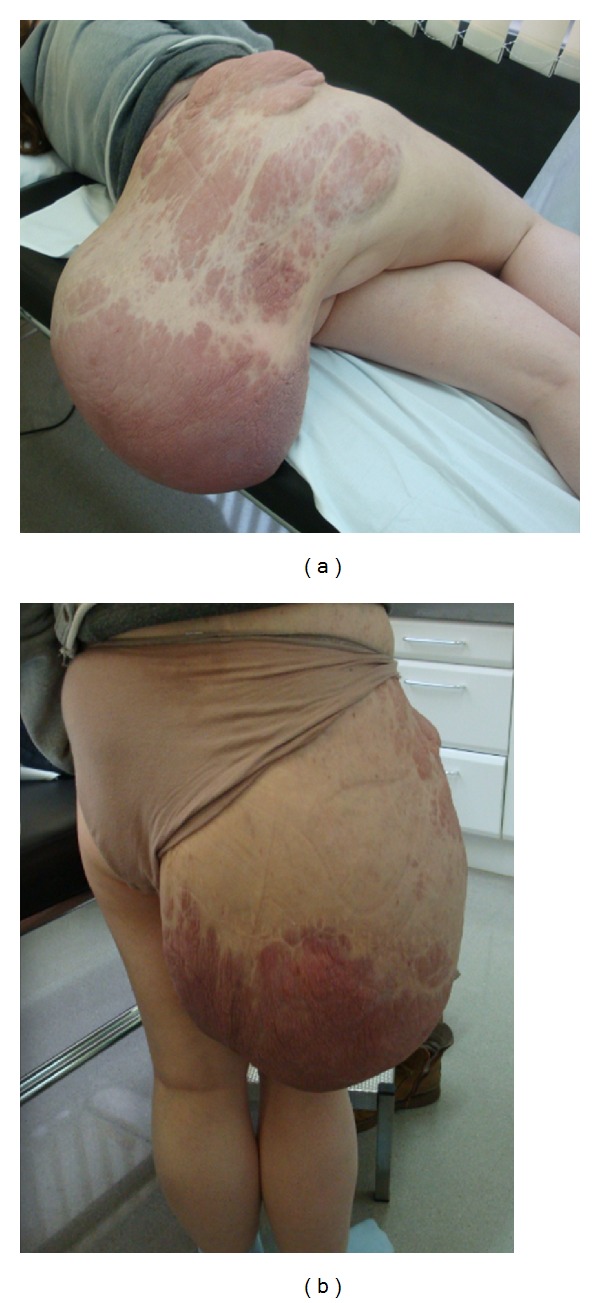
Preoperative clinical images. Mass in the right buttock and lumbar region.

At another treatment facility, when the patient was 18 years of age, she underwent debulking of a tumor from the right buttock, which was complicated with a very profuse intraoperative hemorrhage resulting in a severe hypotension and acute kidney failure requiring 8 units of blood for stabilization. The patient was later discharged and did not continue followup at that treatment facility. In spite of this, the mass in the right buttock and lumbar region continued undergoing slow growth until it limited her daily activities. Upon arriving at our center, physical examination revealed a large, firm, amorphous, fluctuant, soft tissue mass measuring 60 × 50 × 30 cm. that originated from the lumbar region and extended to the gluteal fold and overhanging from the posterior aspect of her thigh (Figure 1). 

**Figure 2:**
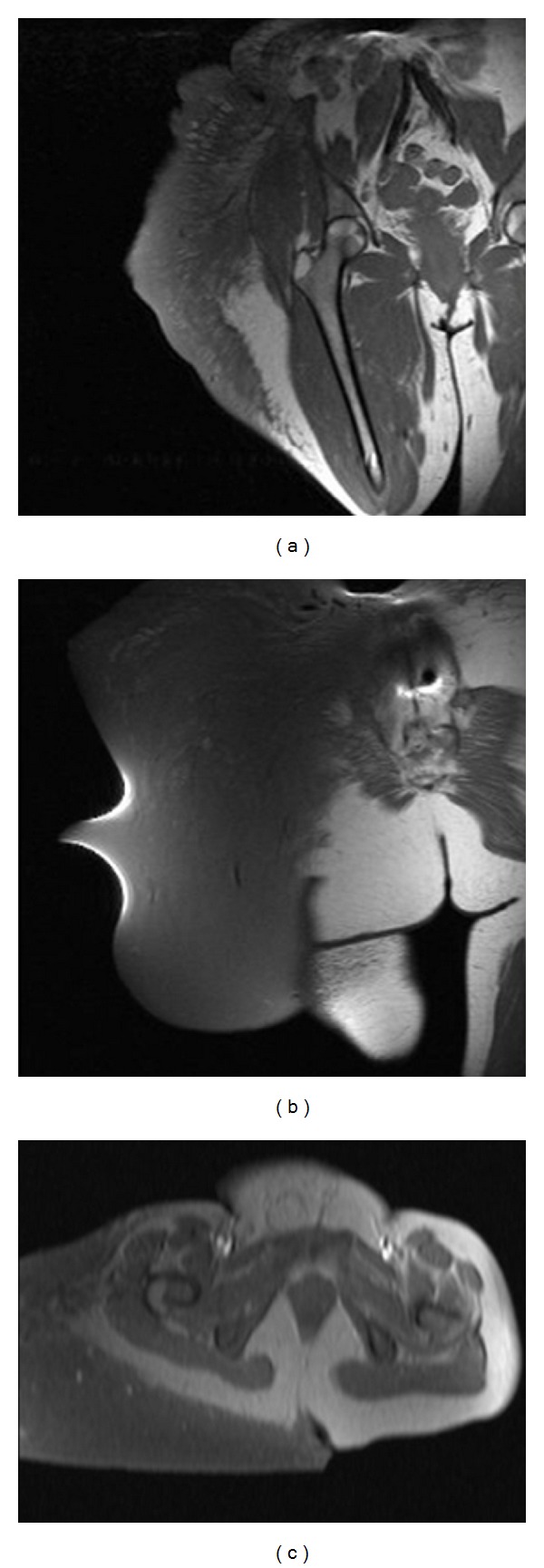
Preoperative MRI images. MRI was technically difficult due to the dimensions of the tumor.

MRI was technically difficult due to the dimensions of the tumor. The extension of the mass was unable to be determined with certainty because portions of it remained outside of the field of view. The mass exhibited intermediate signal on both T1- and T2-weighted images, and several fluid collections were identified (Figure 2). The images demonstrated multiple serpiginous flow voids in keeping with prominent vasculature. The mass did not infiltrate the underlying gluteal musculature. Core needle biopsy was performed and the results were consistent with PNF; there was no evidence of malignancy in the tissue sample.

The case was reviewed at our multidisciplinary musculoskeletal tumor committee. The treatment consensus opted for preoperative embolization, as this would potentially reduce the perioperative blood loss considering the vascularity and size of the lesion. Surgical resection of the bulk of the lesion would then be undertaken and closure achieved with local tissue advancement or free flap transfer. It was not feasible to widely excise the lesion as it was infiltrating almost circumferentially around the patient.

**Figure 3:**
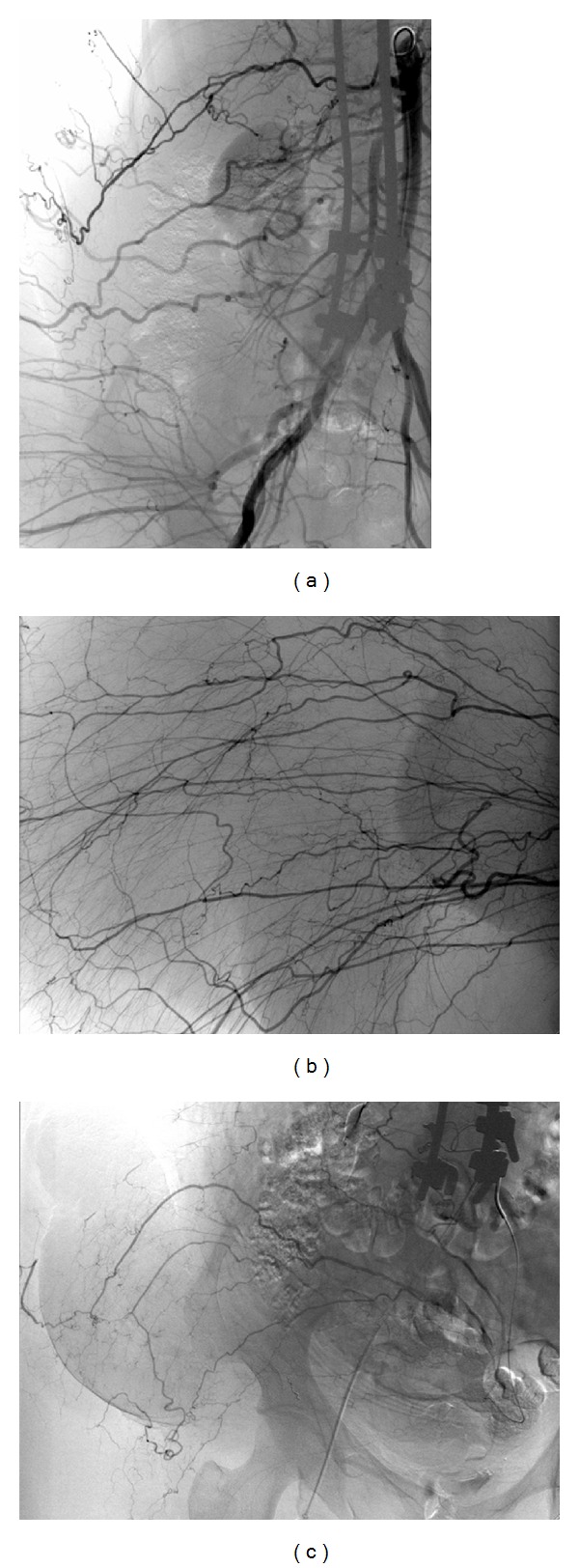
The hypervascularization of the tumor is confirmed by the arterial feed from the ipsilateral intercostal and lumbar arteries (a), ipsilateral gluteal arteries (b), and branches from the contralateral internal iliac artery (c).

**Figure 4:**
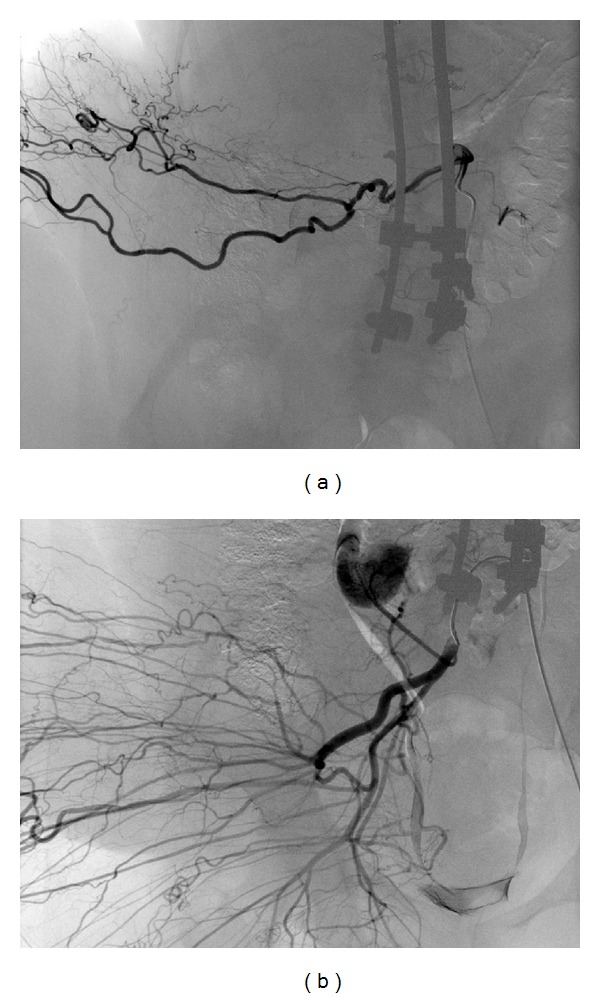
Selective catheterization is performed to do a selective embolization with polyvinyl alcohol particles and also with microcoils in some branches. (a) Lumbar artery. (b) Gluteal artery.

The diagnostic angiogram confirmed the presence of multiple vessels supplying the tumor (Figure 3). The feeding vessels identified were the right intercostal and lumbar arteries (Figure 4), the right superior and inferior gluteal arteries, and the right circumflex iliac artery. Branches of the right profunda femoris and the left internal iliac arteries (Figure 3(c)) were also visualized. A supraselective catheterization was done to perform a selective embolization using Gelfoam and PVA particles. Exceptionally, microcoils were also used. Due to the number and caliber of vessels to treat as well as the amount of contrast that will need to be used, the preoperative embolization was performed sequentially 5 days and 1 day before surgery.

**Figure 5:**
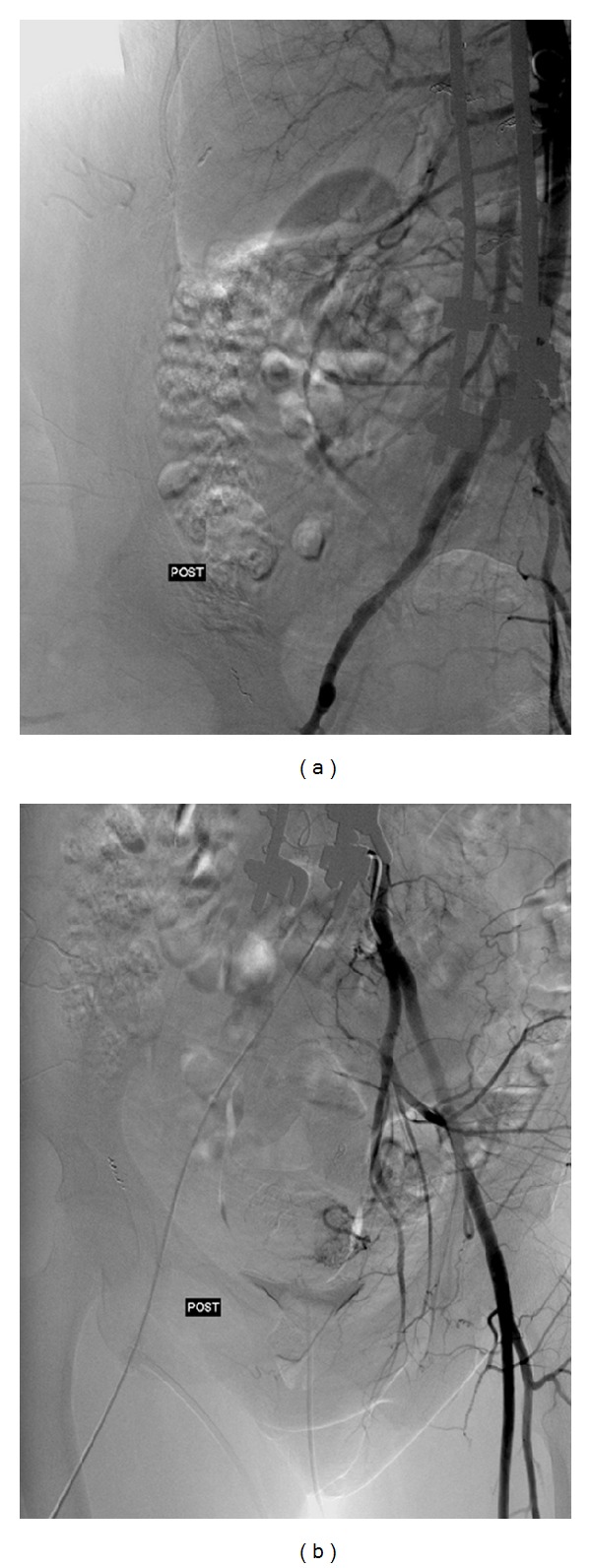
Final angiogram (postembolization). (a) Abdominal aortogram. (b) Left internal iliac artery.

The final angiogram showed almost complete devascularization of the tumor (Figure 5). Tumor regression was satisfactory, with large areas of skin necrosis after embolization and with no associated systemic complications.

**Figure 6:**
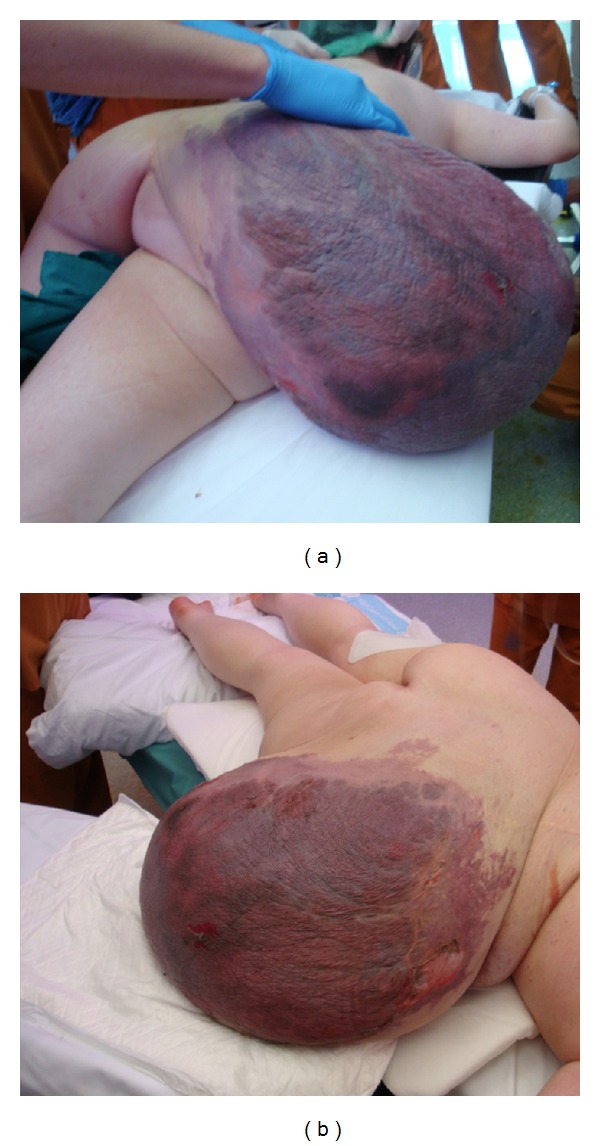
Preoperative clinical images. Large areas of skin necrosis after embolization.

**Figure 7:**
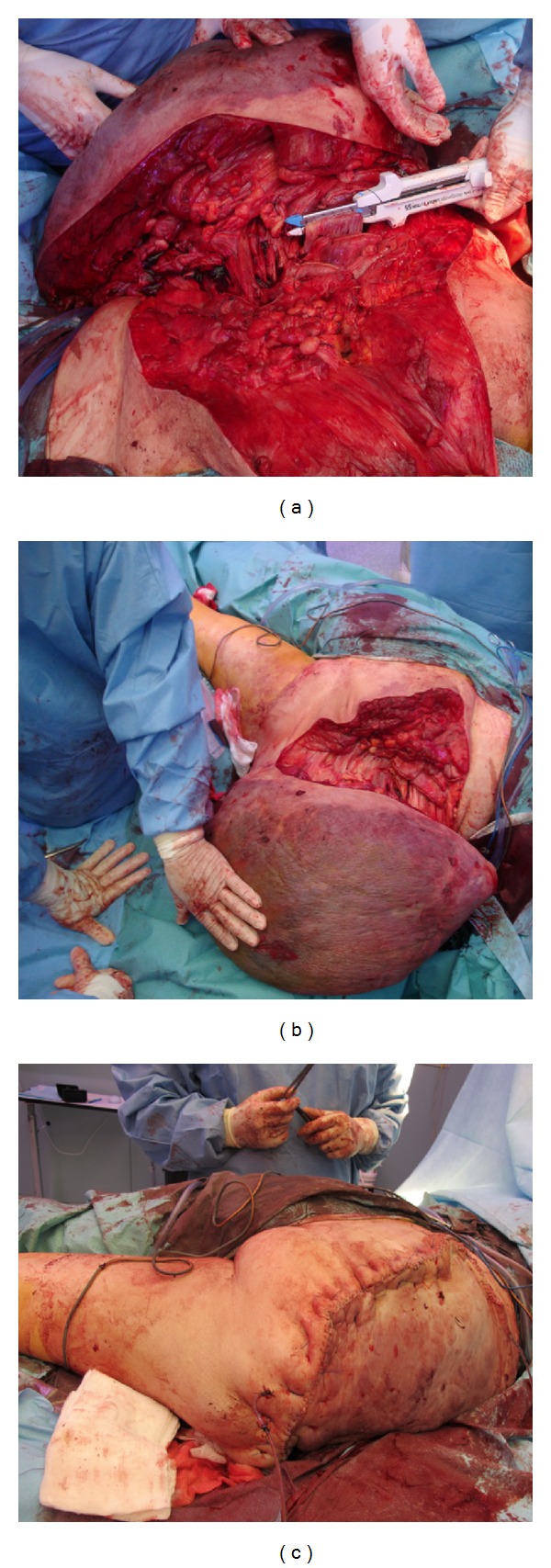
Intraoperative clinical images. (a) The use of the Endo GIA. (b) Tumoral excision. (c) Local skin flap.

Twenty-four hours after the second intravascular embolization, the patient was transferred to the operating room for surgical resection (Figure 6). By positioning her prone on the operating table we were able to manipulate the lesion satisfactorily. An incision was made along the right posterior medial limb from the upper portion of the buttock to the thigh. Dissection through the superficial tissues necessitated some dissection through tumor tissue, which was highly vascular with large friable vessels. To reduce intraoperative bleeding, we decided to use the system Stapling Endo (Endo GIA) (Figure 7(a)). Once deep to the lesion, it was possible to encompass it and dissect it off the deep structures through a normal fatty plane, tying off the main feeding vessels that were demonstrated by angiography. The huge pedunculated gluteal mass was removed completely (Figure 7(b)). Local skin flaps were extensively mobilized and primary closure obtained (Figure 7(c)). Estimated blood loss for the procedure was 1000 mL, and the patient received 4 units of packed red blood cells. There were no intraoperative complications.

**Figure 8:**
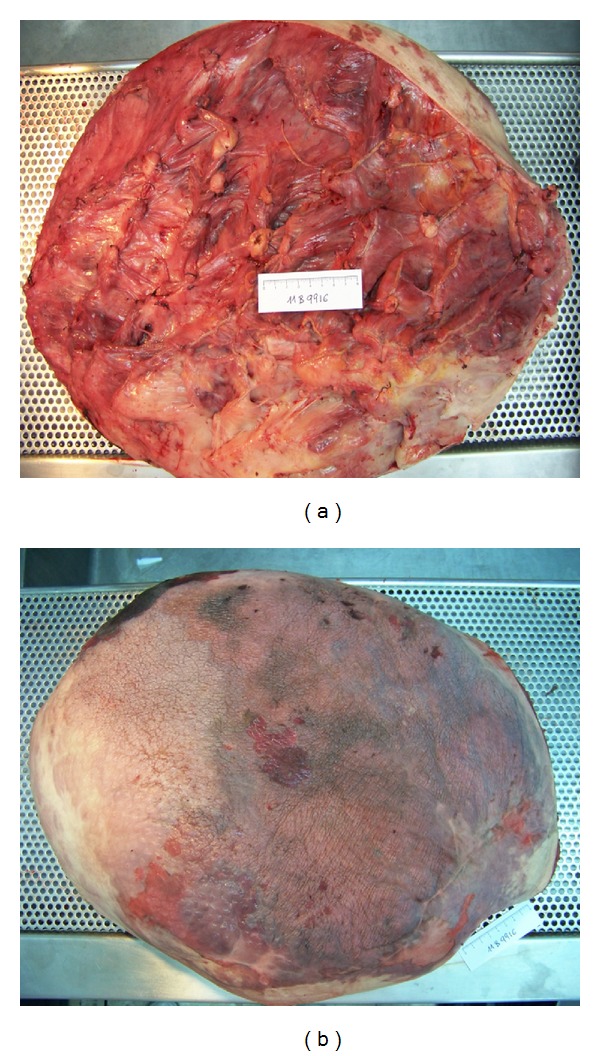
Gross pathology images. Diffuse presence of a myxoid-like white tan tissue, infiltrating into the surrounding fat.

**Figure 9:**
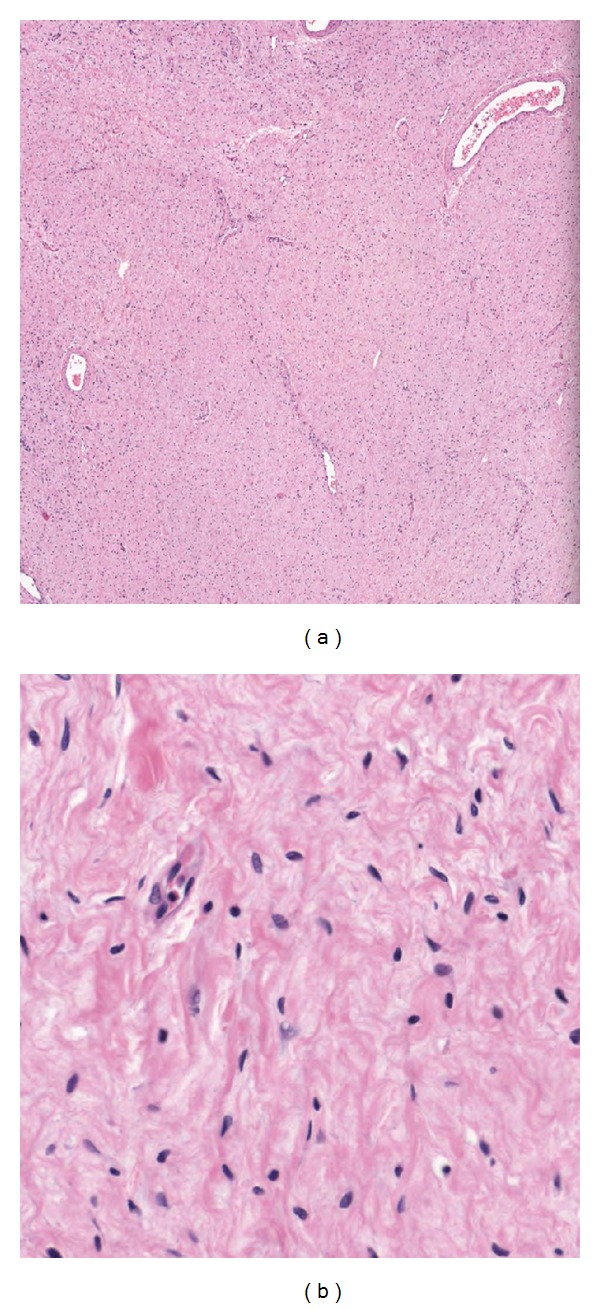
Tumor histology. Plexiform neurofibroma with no evidence of malignancy.

The specimen weighed 16,700 gr. and measured 60 × 45 × 27 cm (Figure 8). Grossly there was diffuse presence of a myxoid-like white tan tissue, infiltrating into the surrounding fat. Histological examination revealed a plexiform neurofibroma with no evidence of malignancy (Figure 9). Superficial margins were positive, consistent with the intralesional procedure.

**Figure 10:**
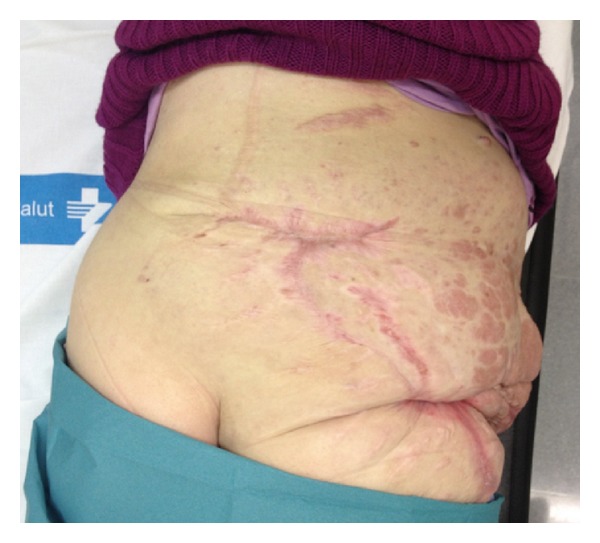
Postoperative (6-month followup) clinical images.

Two weeks after the surgery, the patient had central areas of skin breakdown and cellulitis of the right lower extremity. The patient underwent revision of the closure secondary to infection of the surgical wound. Large cavities containing pus were identified. Cavities were curetted and pulse lavaged. Cultures grew *Escherichia coli* and the patient was started on IV cefepime, vancomycin, and gentamicin. Vacuum-assisted wound closure (VAC; Kinetic Concepts Inc., San Antonio, TX, USA) was placed with 150 mmHg continuous suction and the patient responded well. The surgical wound healed and the patient was discharged after a total of 5 weeks and was followedup in clinic with physical examination and serial local MRI every 3 months (Figure 10).

**Figure 11:**
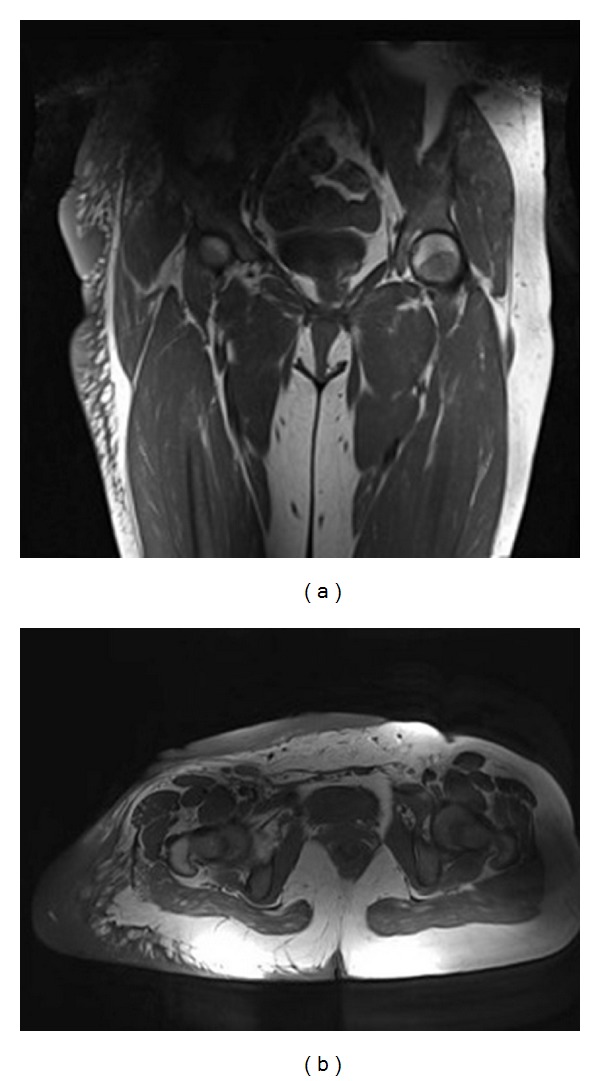
Postoperative MRI images (2-year followup). Not recurrence or tumor progression.

Two years postoperatively, the patient walked without assistance. She was asymptomatic, and physical exam did not reveal any local recurrence. There was considerable improvement in her appearance and mobility. Local MRIs have not revealed recurrence or tumor progression (Figure 11). 

## 3. Discussion

In this paper, we present the treatment with arterial embolization and surgical resection of a patient who developed an invalidating giant PNF involving branches from her right lumbosacral plexus in her right buttock. To our knowledge, this is the first report of a double sequential preoperative intravascular embolization of a giant PNF.

Clinical management for the PNF requires a multidisciplinary approach. However, current treatment options for PNF are limited to surgical intervention. The surgical experience of giant neurofibromas is limited to case reports [11, 19–21]. Resection is performed when the tumor is severely disfiguring or severely compromises functionality [22]. Complete resection is often difficult because of the extensive and infiltrative nature of these lesions [4, 22–24]. In our case, an intralesional procedure was the only possible resection margin because of the almost circumferential nature of the tumor around the patient's body.

Although life-threatening intraoperative hemorrhage in neurofibromas is uncommon, it has been reported [25–31]. It is postulated that these hemorrhages are caused by rupture of friable vasculature secondary to arterial dysplasia or vascular invasion by the neurofibroma [31, 32]. Therefore, the most immediate challenge for surgical management is hemostasis, especially when the dissection is intralesional as in our case. Diathermy is of limited use as the tissue is very friable [31]. A number of authors have reported significant blood loss during surgery requiring high volume transfusion. Taking this into account, we opted to use pre-operative intravascular embolization and the intraoperative use of the Linear Cutting Stapler system (Stapling Endo GIA). This system of automatic mechanical stapling has been described as a feasible technique for muscle transection allowing for a decrease in surgical time and minimizes blood loss with no more complications than standard electrocautery technique [33].

Pre-operative coil embolization was used to reduce the blood flow to the tumor and to limit intraoperative blood loss [29, 34, 35]. The vessels were embolized proximally to reduce the flow of the major vascular pedicles at the surgical dissection plane and to reduce skin necrosis associated with a distal embolization technique. 

The time delay between intravascular embolization and surgery is controversial. It is important to balance between the risk of cutaneous necrosis and the risk of bleeding. In accordance with the plastic surgery department, we decided to wait 5 days between the first embolization and surgery, in order to objectify the effects of preoperative embolization, increase the preoperative knowledge of skin condition, and determine the best choice of postoperative skin coverage. During this time, the patient was rehydrated and readapted for subsequent tumor surgery. Littlewood and Stilwell [35] embolized a plexiform neurofibroma; however, there was a delay of 1 month before surgical excision resulting in significant perioperative blood loss. Jones et al. [34] embolized a giant plexiform neurofibroma prior to surgery, and only four units of packed red cells were required intraoperatively, but the patient had postoperative central areas of skin breakdown that were managed with vacuum-assisted closure dressings.

The management of a tumor of this size is inevitably bound to present a high rate of postoperative complications. A total of 16.7 kg of tumor and fluid was removed from a patient who weighed 75 kg prior to excision. Understandably, the vascularity and lymphatic drainage was altered as a consequence of excision. The postoperative course in these patients is often complicated by lymphedema, cellulitis, and delayed healing [20, 33]. Lymphedema is associated with recurrent tissue infections and nonhealing wounds [39]. Vacuum-assisted wound closure has been shown to improve wound healing and proved useful in this patient [20, 33, 40].

There is a significant discrepancy in the size of tumors that are described as “giant” neurofibromas (Table 1). Some authors reserve this term for tumors weighing more than 20 kg [20], even though no clear consensus has been reached [34]. Reviewing the literature, we have found that all articles published on “giant” neurofibromas are case reports and in many of these articles, the information submitted is incomplete regarding tumor and patient specifications [41–43]. The correlation between the percentage of tumor weight and the total weight of the patient should be taken into account, since the aforementioned management issues occur more frequently in tumors that represent a significant percentage of the overall patients' weight [20]. A more precise definition would facilitate more accurate comparisons between published data; therefore, we feel that a new term to define “giant” neurofibroma is needed. Therefore, and taking into account the published cases of large neurofibromas, we propose that the term “giant neurofibroma” be used for referring to those neurofibromas weighing 20% or more of the patient's total corporal weight. 

## Figures and Tables

**Table 1:** Giant NF cases report revision of the literature.

Author	Sex	Weight	Type NFs	Tumor weight	Tumor size	Tumor/patient	Age
Beall and Vander Kolk, 1997 [8]	Male	—	PNF	32 kg	—	—	26
Salazar et al., 1998 [9]	Male	—	—	6,3 kg	—	—	42
	Male	—	—	—	—	—	65
Margaritora et al., 2002 [10]	Male	—	PNF	—	15 × 15 cm		30
Karaoğlanoğlu et al., 2004 [36]	Female	—	—	—	27 × 19 × 16 cm	—	20
Cebesoy et al., 2007 [11]	Male	—	PNF	—	2 × 3 × 20 cm	—	6
Yang et al., 2009 [12]	Male	—	DNF	—	15 × 11 × 11 cm	—	34
Rallis and Ragiadakou, 2009 [37]	Male	—	PNF	—	—	—	19
Jones et al., 2010 [34]	Male	—	DNF	15 kg	65 × 51 × 10 cm	—	45
Savva et al, 2010 [38]	Male	—	PNF	—	26 cm	—	22
Ji et al., 2011 [19]	Male	—	PNF	—	21 × 17,5 × 5 cm	—	12
Ross et al., 2011 [20]	Male	125 kg	PNF	39 kg	47 cm	31,2%	37
Vélez, 2012	Female	75 kg	PNF	15 kg	60 × 45 × 27 cm	20%	22
